# To explore the pathogenesis of Bell's palsy using diffusion tensor image

**DOI:** 10.1038/s41598-023-42570-8

**Published:** 2023-09-15

**Authors:** Yi Qin, Jihua Liu, Xuening Zhang, Xiaonong Fan, Guiping Li, Yinghui Chang, Li Li

**Affiliations:** 1 Radiology Department, First Teaching Hospital of Tianjin University of Traditional Chinese Medicine, No. 88, ChangLing Road, XiQing District, Tianjin, 300381 China; 2 National Clinical Research Center for Chinese Medicine Acupuncture and Moxibustion, No. 88, ChangLing Road, XiQing District, Tianjin, 300381 China; 3 Radiology Department, The Second Hospital of Tianjin Medical University, No. 23, Pingjiang Road, He Xi District, Tianjin, 300211 China

**Keywords:** Myelin biology and repair, Peripheral nervous system

## Abstract

To explore the pathogenesis of Bell's palsy using the diffusion tensor image on 3.0 T MR. The healthy people and the patients with Bell's palsy underwent intraparotid facial nerve scanning by using the DTI and T1 structural sequence at 3.0 T MR. The raw DTI data were performed affine transformation and nonlinear registration in the common MNI152_T1 space and resampled to the 0.4 mm^3^ voxel size. A group of 4 spherical seed regions were placed on the intratemporal facial nerves in the common space, bilaterally and symmetrically. The DTI data in the common space were used to track the intratemporal facial nerve fibers by using TrackVis and its Diffusion Toolkit. Each tractography was used to construct the maximum probability map (MPM) according to the majority rule. The fractional anisotropy (FA), mean diffusivity (MD), axial diffusivity (AD) and radial diffusivity (RD) were calculated and extracted on the basis of MPM. For healthy people, there was no significant difference in FA, MD, RD and AD of bilateral facial nerves. For patients with Bell's palsy, there was no significant difference in AD, there was significant difference in FA, MD and RD between the affected nerve and the healthy nerve (P < 0.02). This study showed that the myelin sheath injury of the intratemporal facial nerve is the main cause of Bell's palsy. Most neural axons are not damaged. The results may explain the pathogenesis of the Bell's palsy, which is self-limited for most cases.

## Introduction

The most common cases of acute-onset peripheral facial paresis/paralysis are considered Bell's palsy, named after Charles Bell in 1831. Clinical signs of Bell's palsy include loss of facial tone with obliteration of the nasolabial fold, inability to raise the eyebrows and wrinkle the forehead, open/draw the corner of the mouth, and completely close the eye on the affected side. To develop and employ the targeted treatment approaches, it is critical to determine the mechanism of Bell's palsy^1–3^.

Imaging plays an important role in the evaluation of intratemporal facial nerve disorders. High-resolution temporal bone CT, Ultrasound, and MR sequences with gadolinium have been used to evaluate the intratemporal facial nerve with Bell's palsy^4–8^. Diffusion tensor imaging (DTI) with tractography (DTT) on MR has been developed as a new modality to make 3D reconstructions of the facial nerve. By using the DTT, the cisternal and intraparotid segments of the facial nerve were effectively identified, as well as their relationships with tumors^9, 10^. However, there are a few challenges in using DTT to reveal the intratemporal facial nerve, the lesion which may be the main cause of facial nerve palsy.

The intratemporal facial nerve is characterized by a fine and complex anatomical course, which is almost entirely enclosed by the bony facial canal and lack of liquid in it. The purpose of this study is to track and analyze the intratemporal facial nerve of healthy people and patients with Bell's palsy using DTI with tractography. The DTI datasets were acquired with readout segmentation with the navigator-echo correction technique (RESOLVE), which may provide high-resolution diffusion-weighted images and significantly reduce geometric distortions due to susceptibility artifacts^11, 12^.

## Materials and methods

### Subjects

This study was conducted at the First Teaching Hospital of Tianjin University of Traditional Chinese Medicine between April 2019 and September 2019. The authors declare that all subjects participated voluntarily. Informed consent was obtained from all participants in this study. The Declaration of Helsinki was adequately addressed. The study was approved by the IRB of The First Teaching Hospital of Tianjin University (reference number: TYLL2022[Z]No 010).

All volunteers included 18 healthy people and 19 patients. The 18 healthy people with normal facial nerves, including 17 right-handed and 1 left-handed (10 females and 8 males; mean age: 40.9 ± 6.6 years; range: 23–52 years). The 19 patients with unilateral acute-onset peripheral facial paresis/paralysis excluding central nervous system diseases (all right-handed; 13 males and 6 females; mean age: 45.4 ± 15.3 years; range: 23–63 years). There were 13 cases of right facial paresis/paralysis and 6 cases of left facial paresis/paralysis. The onset time of 19 patients is from 1 day to 1 month, and 1 case is 2 years. The house House–Brackmann (H–B) facial nerve grading scale facial nerve function was classified from grade III to VI, which is moderate to severe. The main symptoms were loss of frontal strip on one side and incomplete closure of ipsilateral eyelids. 10 patients had a history of periauricular pain or pharyngeal pain before and after the onset, and 9 patients did not find any discomfort before the onset.

### MR imaging procedures

T1WI and DTI were acquired using a 3.0 Tesla MR scanner (Magnetom Skyra; Siemens Healthcare, Erlangen, Germany) with a 20 channels head-neck matrix coil. The three-dimensional T1 weighted sequence, named 3D-MPRAGE, was performed with the following parameters: voxel size = 1.0 × 1.0 × 1.0 mm, TR = 2000 ms, TE = 1.97 ms, field of view FOV = 256 × 256 mm.

An axial DTI using the RESOLVE technique, which was acquired in 20 directions, with the following parameters: matrix = 220 × 220, field of view = 220 mm × 220 mm; voxel size = 1.2 × 1.2 × 3.0 mm; TR = 4290 ms, TE1 = 71 ms, TE2 = 105 ms; and *b* value = 1000 s/mm^2^. A total of 30 slices were scanned in a scan time of 12 min and 49 s.

## Data post-processing

### T1WI Data post-processing

**Figure 1:**
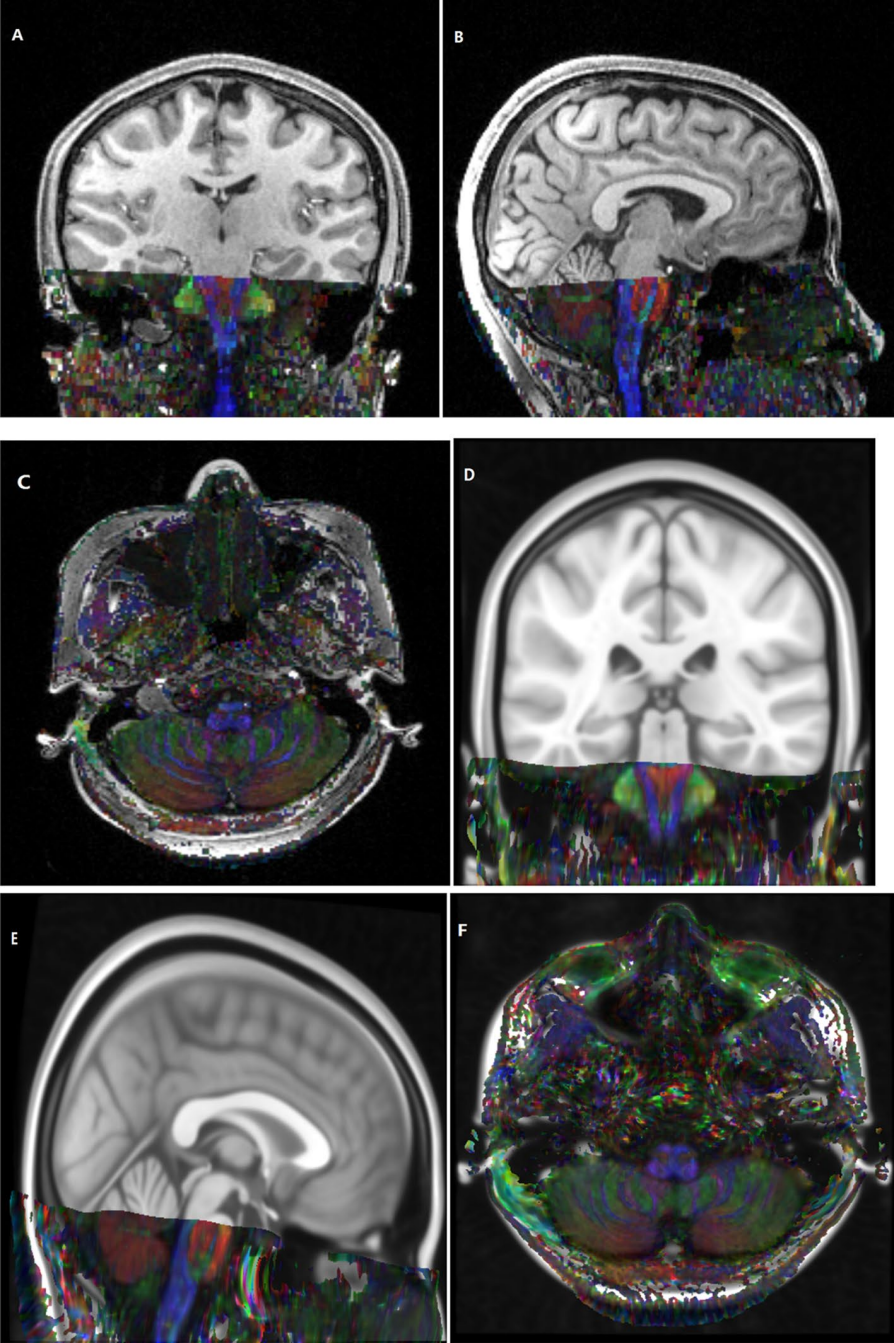
A patient resource FA map with a voxel size of 1.2 mm × 1.2 mm × 3.0 mm was consistent in the common space of MNI152_T1_0.4 mm template with a voxel size of 0.4 mm^3^ by nonlinear registration.

**Figure 2:**
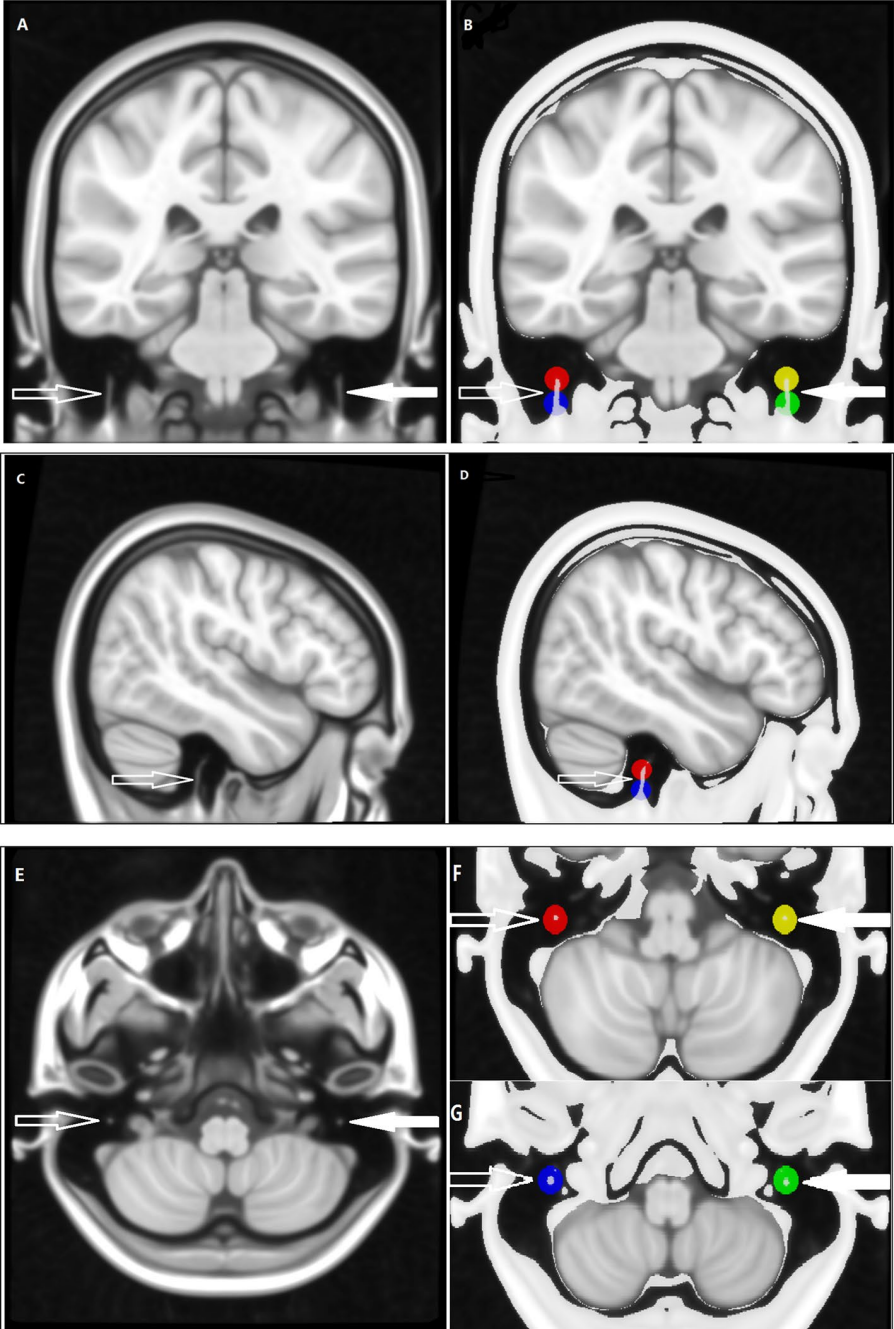
The symmetrically spherical ROIs (right: red and blue; left: yellow and green) with the same diameter of 5 mm, were set on intratemporal facial nerves (right: hollow arrow; left: solid arrow) in the 0.4 mm^3^ voxel MNI common space (named MNI152_T1_0.4 mm).

To construct group-level common template space of 0.4 mm^3^ voxel size, the 0.5 mm^3^ voxel of MNI152_T1_0.5 mm brain template (International Consortium for Brain Mapping) was normalized and resampled to 0.4 mm^3^ template using the "old norm" method of SPM12, which named MNI152_T1_0.4 mm template in the present study (Fig. 1). By using FSL-maths, a group of 4 common spherical seed regions (5 mm in diameter) was calculated and placed on the mastoid segment of intratemporal facial nerves, bilaterally and symmetrically (Fig. 2).

### DTI Data post-processing

**Figure 3:**
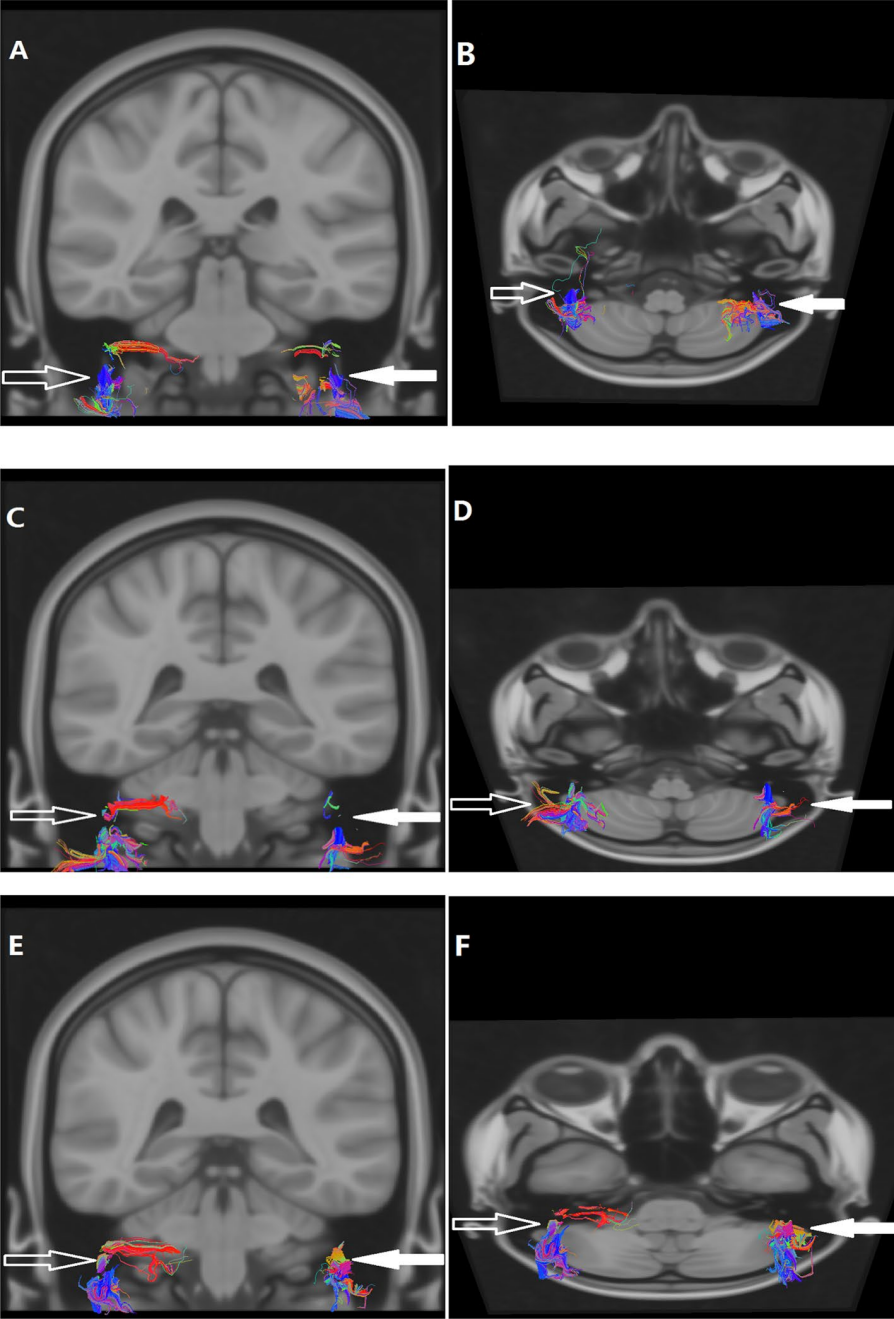
DTI fiber tracing of bilateral intratemporal facial nerves was performed. A patient (**A**, **B**) is a female, 46 years old, with right facial paralysis for 3 days and H-B facial nerve function grade IV. A patient (**C**, **D**) is a male, 23 years old, with right facial paralysis for 7 days and H-B facial nerve function grade III. A patient (**E**, **F**) is a male, 59 years old, with left facial paralysis for 1 day and H-B facial nerve function grade III. The left (solid arrow) and right (hollow arrow) intratemporal facial nerves were indicated by the arrows respectively.

After eddy, current correction of the DTI data, affine transformation, and nonlinear match­ing were performed on the diffusion images matching with the MNI152_T1_0.4 mm template without skull-stripping by using the FSL (Oxford, UK). Then the voxel size of the DTI was resampled to 0.4 mm^3^ in the common space of the MNI152_T1_0.4 mm template. DTI data were used to track each intratemporal facial nerve fiber in 0.4 mm^3^ common space by using TrackVis and its Diffusion Toolkit (DTK). The intratemporal facial nerve fibers were traced with a thresh­old value of FA > 0.15 and an angle threshold value of 60°. To reduce errors, the step length of fibers less than 2 mm was discarded (Fig. 3).

**Figure 4:**
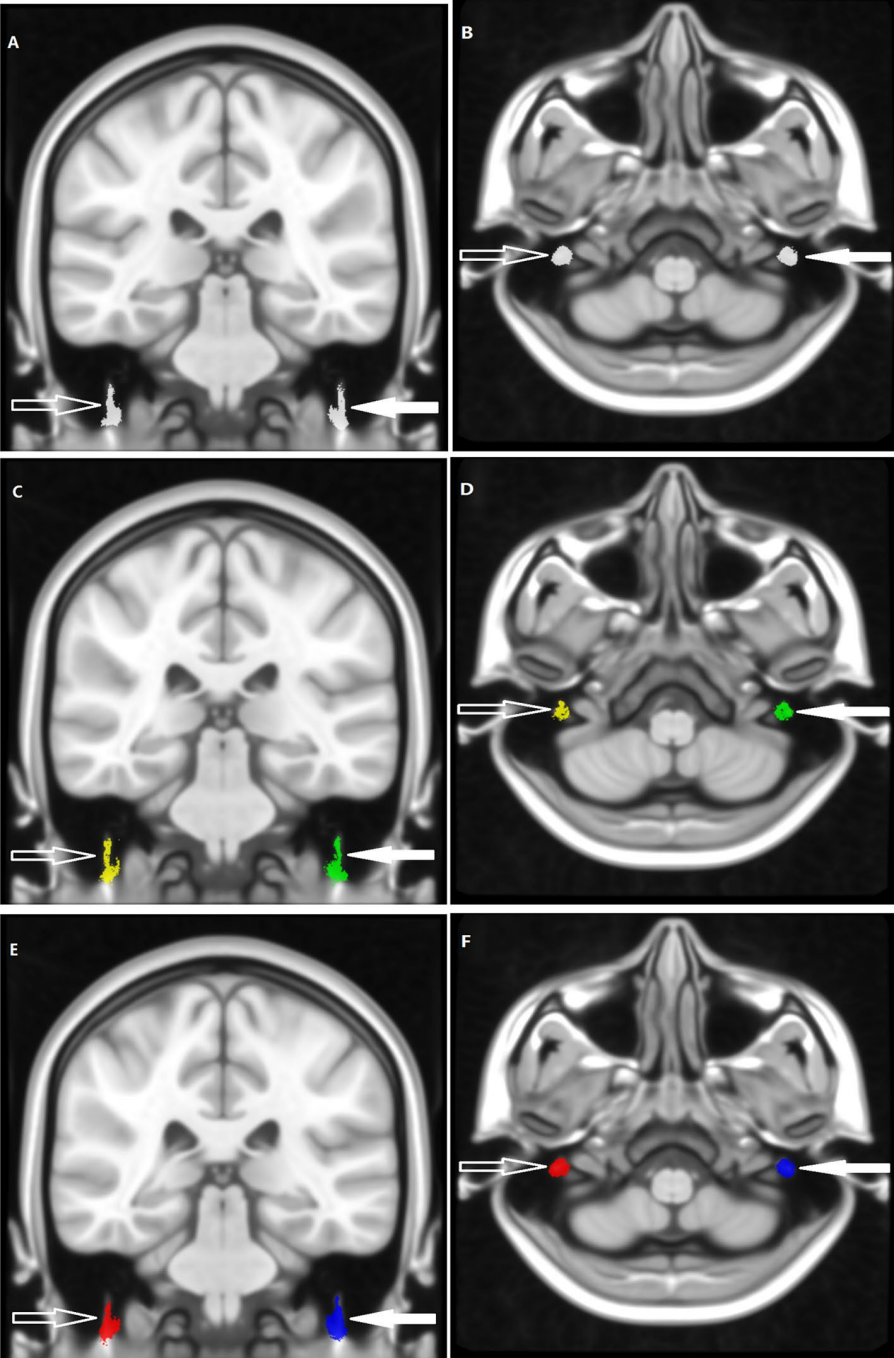
According to the majority rule across subjects, the MPM of bilateral intratemporal facial nerves was constructed. The MPM of the healthy people (**a**, **b**), the patients (**c**, **d**), all people including the healthy people and patients (**e**, **f**). The left (solid arrow) and right (hollow arrow) intratemporal facial nerves were indicated by the arrows respectively.

All results then were used to construct the maximum probability map (MPM), which assigned the most probable labels to each voxel according to the majority rule across subjects (Fig. 4). The indexes of DTI, named fractional anisotropy (FA), mean diffusivity (MD), axial diffusivity (AD), and radial diffusivity (RD), were calculated and extracted based on MPM.

In the present study, the MPM of 18 healthy people was named 18-Health-MPM, the MPM of 19 patients people was named 19-Patient-MPM, and the MPM of all 18 healthy people and 19 patients were named 37-All-MPM, respectively.

### Statistical analysis

SPSS18 (Chicago, Illinois, USA) was used for statistical analysis. To conduct a comprehensive analysis of facial nerve, this study collected DTI data of the left/right and the affected/healthy side facial nerve and then conducted descriptive statistics and normal tests on the data. For quantitative measurement, paired and unpaired t-test was used to evaluate the FA, MD, AD, and RD values. All statistical significance tests were two-tailed hypotheses, with P < 0.05 as statistically significant.

## Results

All DTI indexes of left/right and affected/healthy side intratemporal facial nerve accorded with a normal distribution (K-S test, P > 0.05).

### Base on 18-Health-MPM

There was no significant difference in FA, MD, RD, and AD of the left and right intratemporal facial nerve (Table 1).

**Table 1:** DTI indexes of bilateral facial nerve in healthy people (mean ± SD).

	Left side	Right side	*P* value
**FA**			
FA(18-Health-MPM)	0.303 ± 0.069	0.291 ± 0.068	0.382 (t = 0.897)
FA(37-All-MPM)	0.305 ± 0.072	0.292 ± 0.069	0.352 (t = 0.957)
**MD**			
MD(18-Health-MPM)	1.066 ± 0.1885	1.124 ± 0.1869	0.070 (t = − 1.933)
MD(37-All-MPM)	1.005 ± 0.152	1.097 ± 0.214	0.094 (t = − 1.775)
**AD**			
AD(18-Health-MPM)	0.00121 ± 0.00014	0.00126 ± 0.00015	0.275 (t = − 1.128)
AD(37-All-MPM)	0.00120 ± 0.00015	0.00128 ± 0.00020	0.397 (t = − 0.869)
**RD**			
RD(18-Health-MPM)	0.00079 ± 0.00012	0.00084 ± 0.00014	0.190 (t = − 1.367)
RD(37-All-MPM)	0.00079 ± 0.00012	0.00089 ± 0.00018	0.275 (t = − 1.127)

MD—the unit is 10^−3^ mm^2^/s.

### Base on 19-Patient-MPM

There was a significant difference in FA, MD, and RD between the affected nerve and the healthy nerve (P < 0.02); there was no significant difference in AD. The FA of the affected nerve was lower than that of the healthy nerve, and the MD and RD were higher than that of the healthy nerve (Table 2).

**Table 2:** DTI index analysis of affected facial nerve and the healthy nerve in patients (mean ± SD).

	Healthy side (N = 19)	Affected side (N = 19)	*P* value
**FA**			
FA(19-Patient-MPM)	0.311 ± 0.053	0.281 ± 0.069	0.011 (t = 2.832)
FA(37-All-MPM)	0.309 ± 0.050	0.278 ± 0.067	0.011 (t = 2.832)
**MD**			
MD(19-Patient-MPM)	1.005 ± 0.152	1.084 ± 0.212	0.019 (t = 0.019)
MD(37-All-MPM)	1.005 ± 0.152	1.097 ± 0.214	0.023 (t = − 2.487)
**AD**			
AD(19-Patient-MPM)	0.00120 ± 0.00014	0.00126 ± 0.00019	0.114 (t = 0.114)
AD(37-All-MPM)	0.00120 ± 0.00015	0.00128 ± 0.00020	0.130 (t = − 1.586)
**RD**			
RD(19-Patient-MPM)	0.00078 ± 0.00012	0.00088 ± 0.00018	0.010 (t = 0.010)
RD(37-All-MPM)	0.00079 ± 0.00012	0.00089 ± 0.00018	0.012 (t = − 2.788)

MD—the unit is 10^−3^ mm^2^/s.

### Base on 37-All-MPM

In 18 healthy people, there was no significant difference in all DTI indexes between the left and right intratemporal facial nerves (Table 1). In 19 patients, there were significant differences in FA, MD, and RD between the affected nerve and the healthy nerve; there was no significant difference in AD (Table 2). Compared with the 19 patients and the 18 healthy people: there was no significant difference in all DTI indexes between the healthy nerves of patients (19 nerves) and the nerve of healthy people (36 nerves) (Table 3). There was a significant difference between RD of the affected nerve and the healthy nerve (including the 19 healthy facial nerves of patients and the 36 bilateral nerves of healthy people, a total of 55); there was no significant difference between FA, MD, and AD (Table 3).

**Table 3:** Base on 37-All-MPM, DTI index analysis of the healthy/affected facial nerves of 19 patients and the healthy nerves (mean ± SD).

	Patients	Healthy nerves	*P* value
FA	0.309 ± 0.050 (N1 = 19)	0.299 ± 0.070 (N3 = 36)	0.593 (t = 0.538)
	0.278 ± 0.067 (N2 = 19)	0.302 ± 0.063 (N4 = 55)	0.157 (t = − 1.430)
MD	1.005 ± 0.152 (N1 = 19)	1.097 ± 0.214 (N3 = 36)	0.092 (t = − 1.714)
	1.097 ± 0.214 (N2 = 19)	1.061 ± 0.178 (N4 = 55)	0.477 (t = 0.715)
AD	0.00120 ± 0.00015 (N1 = 19)	0.00122 ± 0.00015 (N3 = 36)	0.800 (t = − 0.255)
	0.00128 ± 0.00020 (N2 = 19)	0.00128 ± 0.00020 (N4 = 55)	0.130 (t = 1.532)
RD	0.00079 ± 0.00012 (N1 = 19)	0.00080 ± 0.00013 (N3 = 36)	0.777 (t = − 0.285)
	0.00089 ± 0.00018 (N2 = 19)	0.00080 ± 0.00012 (N4 = 55)	0.015 (t = 2.480)

MD—the unit is 10^−3^ mm^2^/s. N1 refers to the number of nerves on the healthy side of the patient. N2 refers to the number of nerves on the affected side of the patient. N3 refers to the number of nerves on both sides of healthy people. N4 refers to the number of nerves on both sides of healthy people plus the number of nerves on the healthy side of patients.

## Discussion

The intratemporal facial nerve courses through the bony facial nerve canal in the temporal bone. The labyrinthine segment, which has been measured on average below 0.7 mm, is the narrowest portion of the canal. That the bony canal confines the expansion of the facial nerve caused by edema or inflammation has been speculated as the etiology of Bell's palsy, especially in the labyrinthine segment^13^. However, there is no direct evidence for the pathogenesis of Bell's palsy.

DTI can non-invasively describe the course of nerve fibers by the degree of restriction of free movement of water molecules. Specifically, the integrity of the axon and the myelin sheath of nerve fibers limits the free diffusion of water molecules. The higher the FA the higher integrity of nerve fibers, nevertheless, the damage of the myelin sheath of nerve fibers will lead to the decrease of FA. MD indicates the ability of water molecules to diffuse in all directions of motion. The nerve damage will lead to faster diffusion of water molecules and higher MD^14^. AD and RD can accurately determine the specific cause of diffusion differences. It is generally believed that the increase in AD represents axonal injury of nerve fibers, and the increase in RD indicates the myelin damage of nerve fibers^15^.

The RESOLVE sequence is a sort of multi-shot echo planar imaging (ms-EPI) technique based on the read-out segmentation with a navigator echo correction approach. The RESOLVE technique can provide high signal-to-noise ratio images and significantly reduce susceptibility-based distortions compared with the conventional single-shot DTI technique. For the intratemporal facial nerve, which is involved with both air and bone-tissue interfaces, the RESOLVE technique may provide a high-quality and spatial resolution of the nerve. Recently, on ultra-high field MR 9.4 Tesla, intratemporal facial nerve in a cadaveric specimen was performed DTI with a voxel size of 0.4 mm^3^ using an extremely long scan time of approximately 15 h^16^. The tractography was not performed on the facial nerve for its embalmed treatments. To the best of our knowledge, DTI and its tractography of intratemporal facial nerve have not been performed in vivo.

In this study, the raw RESOLVE data were resampled to 0.4 mm^3^ voxel size by performing affine transformation and nonlinear registration in the common MNI152_T1_0.4 mm space (Fig. 1). The resampled RESOLVE data were used to track the intratemporal facial nerve fibers of each subject in the 0.4 mm^3^ group-level common space, and the MPMs of the different group were obtained (Fig. 4). To observe the microscopic differences of intratemporal facial nerve fibers, the DTI indexes based on the different MPMs nerve were extracted.

The results showed that there was no significant difference in DTI indexes between the bilateral nerves of healthy people. There was a significant difference in FA, MD, and RD between the affected nerve and healthy nerve; there was no significant difference in AD. The decrease in FA and the increase in MD suggest that the integrity of the affected facial nerve is damaged. There was no significant difference in AD between affected and healthy facial nerves, suggesting that the axon of the facial nerve was not damaged. The increase in RD suggests that the cause of facial nerve damage is myelin injury.

## Conclusion

This study showed that the myelin sheath injury of the intratemporal facial nerve is the main cause of Bell's palsy. As far as we know, the myelin sheath of the nerve is relatively easy to recover while the axon of the nerve is difficult to regenerate. This study may explain why most of Bell's palsy is self-limited.

## Data Availability

The datasets used and/or analyzed during the current study are available from the corresponding author upon reasonable request.
